# Life with Bacterial Secretion Systems

**DOI:** 10.1371/journal.ppat.1005562

**Published:** 2016-08-11

**Authors:** Tomoko Kubori

**Affiliations:** Research Institute for Microbial Diseases, Osaka University, Suita, Osaka, Japan; The Fox Chase Cancer Center, UNITED STATES

Mysterious organelles that span the bacterial envelope have grabbed my heart throughout my life. It was not a coincidence that bacterial secretion systems, the pivotal machinery delivering virulent determinants into infected host cells, turned out to be my central interest after switching research subjects from the bacterial flagellum, which is a locomotive organelle. The virulent type III secretion systems (T3SSs) and the flagellar secretion systems are now understood to be evolutionarily related.

I started my scientific career as a technician in a lab, conducting structural and functional research on the flagellum of the food poisoning bacterium, *Salmonella typhimurium*. Supervised by two different types of bosses, Keiichi Namba and Chi (Shin-ichi) Aizawa, I learned a lot about how scientific research should proceed. The open and harmonious atmosphere in the lab made me fully enjoy the research. The turning point came early, when I observed *Salmonella* by transmission electron microscopy. I found unique structures on the bacterial surface: needle-like protrusions. During a period of four years when I got my PhD on a different subject in the transcription field, that view of the needles never left my mind. I was young and naïve enough to believe that there was no way that such a beautiful structure couldn’t have an important biological role. The isolated needle complex appeared to be a tiny machine resembling a syringe with a needle attached. It was simply beautiful.

I went back to Chi’s lab at Teikyo University as a postdoctoral fellow and restarted purifying the needle complex in order to determine the component proteins. At that time, genetic evidence had accumulated from many groups suggesting the existence of the T3SSs in pathogenic bacterial species including *Salmonella*, and, importantly, several genes expected to encode component proteins of the T3SS showed similarity in amino acid sequences with flagellar genes. During this period, I had a fateful encounter with Bob (Robert) Macnab, professor in the Department of Molecular Biophysics and Biochemistry (MB&B) at Yale University. One beautiful day, sitting on a lawn somewhere in Japan, I asked him to give me his opinions about my hypothesis that the needle complex was the T3SS itself. I had been totally absorbed by this idea, as I found that the assumption did not have any inconsistencies with the available data. Bob said, “Life is not that easy.” I knew that Bob was not a person who supported a speculative idea without any experimental evidence. I have never been directly under Bob’s supervision, but I now think that he has been among my most important mentors. I was strongly affected by his philosophy during the most sensitive period on building my own style of research. After a couple of years of struggling, a student of mine finally got the amino acid sequence of a component protein and said, “It is InvG.” I still recall that as the happiest moment in my life. InvG was an expected gene product of the T3SS.

I don’t think that I “discovered” the T3SS. I just unveiled its physical entity. But I felt that the direction of research on bacterial secretion systems somehow changed after that. Many laboratories started to work on the structural and functional aspects of the secretion systems of various bacteria. Now, the detailed structures of the T3SSs are mostly solved in high resolution through the efforts of many researchers.

During my second postdoctoral period at Yale University School of Medicine, my interests expanded to the bacterial “effector proteins,” which are the transport substrates of the secretion systems and key players in causing diseases. My advisor, Jorge Galan, professor and chair of the Department of Microbial Pathogenesis, proposed several fascinating projects to me. It was exciting to know how bacteria utilize host cellular systems for their own benefit and to understand the ingenious role of the effector proteins. It was wonderful that most of my projects were successfully completed in five years. I really appreciate the fact that this success was brought about by judicious and insightful advice from Jorge and by extensive discussion with talented lab mates, including Erec Stebbins.

After returning to Japan, I switched my research interest to the type IV secretion systems (T4SSs), which are evolutionarily related to bacterial conjugation systems, and their substrate effector proteins. I simply wanted to start something new. *Legionella pneumophila*, a T4SS-possessing bacterium that infects human lungs and causes pneumonia, was mysterious and therefore an attractive target for me. (Later I found it to be a stubborn organism which never wants to expose its identity to host cells or to researchers.) With my respected partner Hiroki Nagai, I started several projects both on the T4SS structure and on the effector proteins at the Research Institute for Microbial Diseases at Osaka University. We identified many effector proteins and presented the concept of a “metaeffector”; an effector that can regulate another effector. I believe that this was a breakthrough finding, showing the multitiered network in effector proteins for the first time. After many years, we have finally gotten the native structure of the core complex of *Legionella* T4SS. But this is just the beginning to understanding the molecular mechanism of action of T4SSs using the structural analysis.

After more than 20 years of research, I am still mainly working at the bench and publishing papers as a first author, probably because I love experimental research and I am also less ambitious about job status than other people. Despite this matter, my days of scientific experiments are filled with joy and excitement. Science is collaboration between humans and nature. What we do as scientists is translating nature’s voice into human language that we can understand. I try not to miss the faint whispers from the bacteria, as they are providing important messages to us. What Bob said to me after achieving the identification the T3SS is a belief of mine; “Chance favors a prepared mind,” which was originally stated by Louis Pasteur.

**Image 1:**
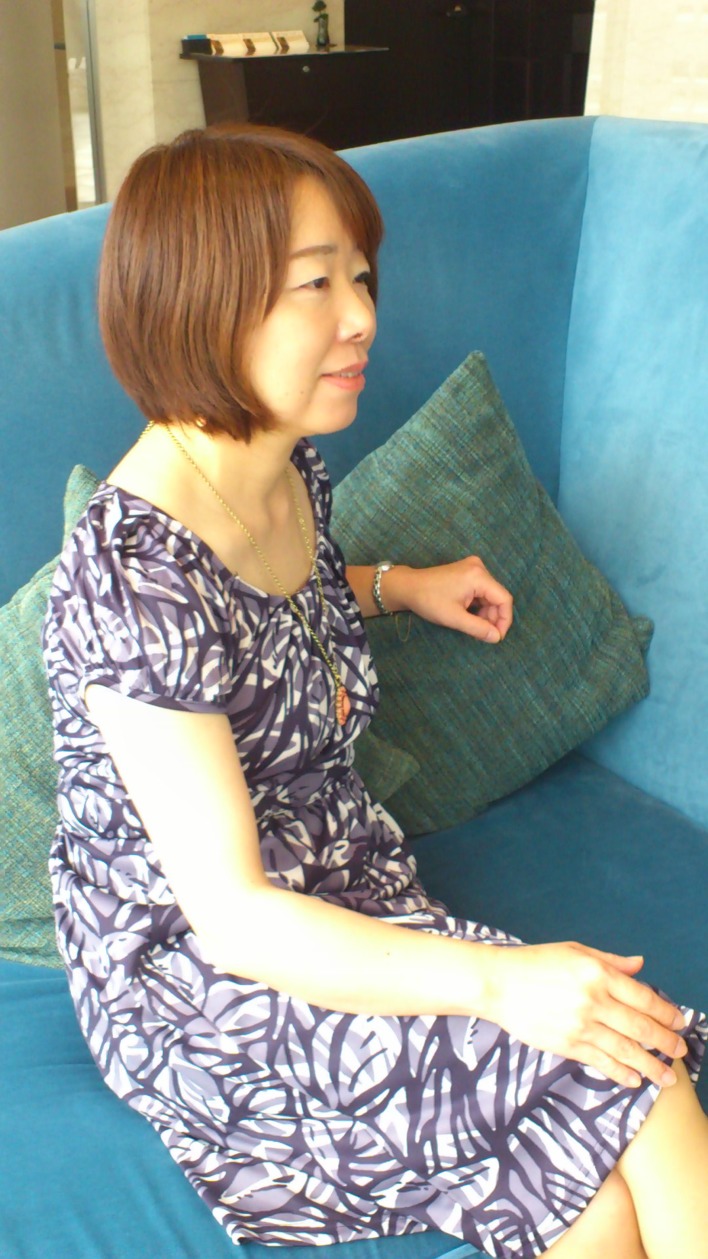
Tomoko Kubori.

